# Green TLC-densitometric method for determination of cytarabine and granisetron in spiked human plasma: optimization and whiteness assessment

**DOI:** 10.1186/s13065-026-01821-1

**Published:** 2026-05-19

**Authors:** Salwa I. Tohamy, Eglal A. Abdelaleem, Adel Lashien, Mahmoud M. Amin, Nessreen S. Abdelhamid

**Affiliations:** 1 Pharmaceutical Analytical Chemistry Department, Faculty of Pharmacy, Beni-Suef University, Beni-Suef, 62514 Egypt; 2 Pharmaceutical Analytical Chemistry Department, Faculty of Pharmacy, Nahda University, Sharq El Nile, Beni-Suef, 62511 Egypt; 3 Pharmaceutical Chemistry Department, Faculty of Pharmacy, Nile Valley University Egypt, Fayoum, 63518 Egypt

**Keywords:** Cytarabine, Granisetron, TLC-densitometry, Human plasma, Green analytical chemistry, White analytical chemistry

## Abstract

Cytarabine (CYT) is a cornerstone in the treatment of acute myeloid leukemia (AML) and is often co-administered with granisetron (GRS) to manage chemotherapy-induced nausea and vomiting (CINV). A precise and sensitive thin-layer chromatographic (TLC) method was developed and validated for the simultaneous determination of CYT and GRS in spiked human plasma samples. Chromatographic separation was achieved by using a developing system consisting of ethyl acetate, ethanol, and ammonia (3:6.8:0.2, by volume), and detection was performed at 240 nm. The method exhibited linearity over the concentration ranges of 0.5–3.0 µg/band for the pure samples and 0.5–3.3 µg/band in spiked plasma for the analytes. The proposed method was validated in accordance with the guidelines of the U.S. Food and Drug Administration (FDA). Its environmental sustainability was evaluated using the Modified Green Analytical Procedure Index, Analytical Greenness Calculator, and Multicolor Assessment Index, demonstrating strong performance with minor areas for improvement. Notably, this study presents the first green TLC method for the simultaneous determination of CYT and GRS in spiked human plasma, offering a valuable tool for therapeutic drug monitoring during chemotherapy.

**Supplementary Information:**

The online version contains supplementary material available at 10.1186/s13065-026-01821-1.

## Introduction

Acute myeloid leukemia (AML) is the most common form of acute leukemia in adults, and cytarabine (CYT) has long been the cornerstone of standard AML treatment protocols [1]. Chemotherapy-induced nausea and vomiting are among the most distressing side effects of cytotoxic chemotherapy, and if not properly managed, they can severely affect patients’ nutrition, well-being, and willingness to continue treatment. Poor management of these symptoms may compromise therapeutic outcomes and increase morbidity, mortality, and healthcare costs [2, 3]. To mitigate these adverse effects, antiemetic agents such as granisetron (GRS) are frequently co-administered with cytotoxic drugs. In clinical practice, intravenous administration of multiple agents, including CYT and GRS, is often required to ensure treatment tolerability and improve patient compliance during cancer therapy, despite the absence of a fixed-dose combination formulation [4].

Cytarabine (CYT), chemically known as cytosine arabinoside (β-D-arabinofuranosylcytosine, ara-C), is a pyrimidine nucleoside analog primarily used to treat acute myeloid leukemia (AML) and non-Hodgkin’s lymphoma [1]. CYT is intracellularly metabolized to its active triphosphate form, which exerts cytotoxic activity through multiple mechanisms, including inhibition of DNA synthesis via suppression of DNA polymerase β and incorporation into DNA, ultimately impairing DNA repair [5].

**Fig. 1:**
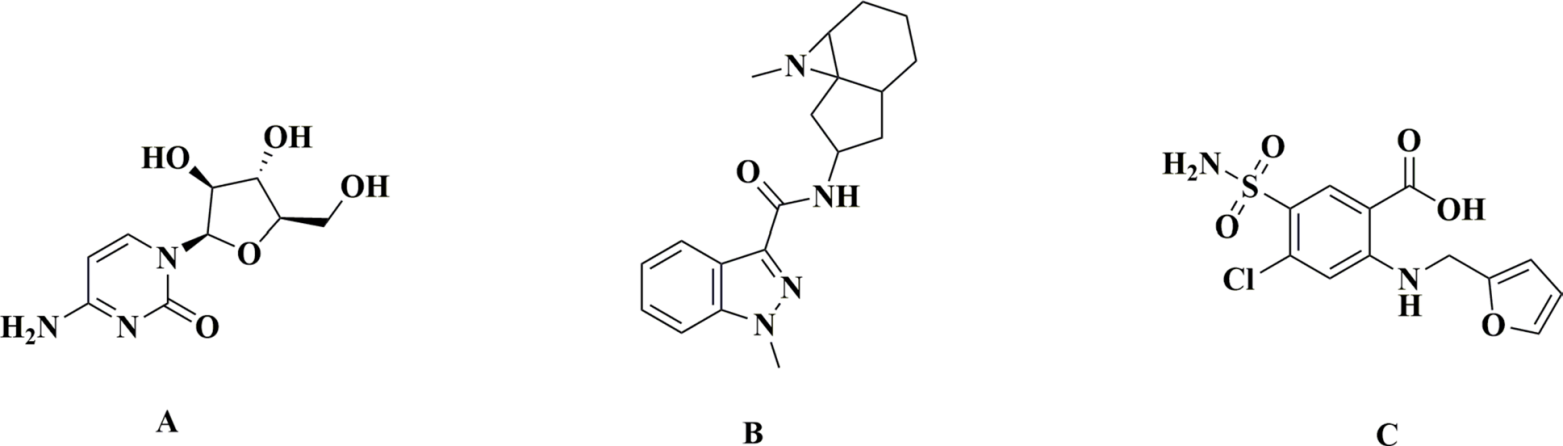
Chemical structure of **A** Cytarabine, **B** Granisetron, and **C** Furosemide

Granisetron (GRS), chemically designated as 1-methyl-N-[(1R,3r,5 S)−9-methyl-9-azabicyclo(3.3.1)non-3-yl]−1 H-indazole-3-carboxamide, is a selective 5-hydroxytryptamine type 3 (5-HT₃) receptor antagonist widely used for the prevention and treatment of chemotherapy-induced nausea and vomiting [6]. It has demonstrated high efficacy and tolerability in both adult and pediatric patients [7]. The chemical structures of CYT and GRS are shown in Fig. 1.

A review of the literature revealed that cytarabine (CYT) has been extensively determined alone or in combination with other drugs using spectrophotometric methods [5, 8, 9] and high-performance liquid chromatography (HPLC) techniques [10–22]. Similarly, granisetron (GRS) has been quantified using various spectrophotometric methods [6, 23–25] and HPLC approaches [7, 26–36]. However, to the best of our knowledge, no analytical method has been reported for the simultaneous determination of CYT and GRS in spiked human plasma. Accordingly, this study aimed to develop and validate a thin-layer chromatographic (TLC) method. The proposed approach offers a sensitive, accurate, and cost-effective tool for therapeutic drug monitoring and bioanalytical applications. Furthermore, its environmental sustainability was confirmed using modern analytical and greenness assessment tools.

## Experimental

### Instrument

Chromatographic analysis was performed using TLC aluminum plates (20 × 20 cm) of 0.25 mm thickness, pre-coated with silica gel 60 F_254_ (Merck, Germany). Samples and standards were applied as 6 mm bands using a CAMAG Linomat 5 autosampler equipped with a 100 µL syringe. The application was performed at a constant rate of 150 nL/s with a 10 mm distance between the bands. The first band was positioned 15 mm from the left edge and 10 mm from the bottom.

After development, the plates were air-dried for 5 min at room temperature. Densitometric scanning was performed at 240 nm in the absorbance mode using a CAMAG TLC Scanner 3 operated by winCATS software. A deuterium lamp was used as the radiation source for the experiment. The scanning speed was set at 20 mm/s with a data resolution of 100 μm/step, and the slit dimensions were adjusted to 5.00 mm ×0.45 mm. All chromatograms were integrated and processed to ensure the accurate quantification of CYT and GRS.

### Materials and reagents

#### Pure sample

Authentic granisetron (GRS, purity 99.87%) was obtained from Rameda Company (Cairo, Egypt), and cytarabine (CYT, purity 99.67%) was purchased from Sigma-Aldrich Chemie GmbH (Germany). Furosemide (FUR), used as an internal standard (purity 99.87%), was supplied by Pharco B Pharmaceuticals (Cairo, Egypt). Human plasma samples were provided by the Holding Company for Biological Products and Vaccines (VACSERA, Cairo, Egypt).

## Chemicals and solvents

All chemicals used in the analysis were of HPLC grade and included methanol, ethyl acetate, and ethanol (Sigma-Aldrich, Germany). Ammonia was obtained from El Nasr Pharmaceutical Chemicals (Abu Zaabal, Cairo, Egypt).

### Optimization of experimental conditions

To maximize the sensitivity and stability of the proposed analytical method, various experimental parameters were systematically optimized, including mobile phase composition, scanning wavelength, and choice of internal standard. Detailed results and discussion of these optimization studies are presented in Sect. "Results and Discussion".

### Standard solutions

Stock standard solutions of CYT, GRS, and FUR were prepared at a concentration of 1000 µg/mL in methanol. Working standard solutions of CYT, GRS, and FUR (100 µg/mL) were freshly prepared by appropriate dilution of the stock solutions.

### Blank plasma samples

Protein precipitation was performed to remove plasma proteins that could interfere with chromatographic separation and densitometric analysis. A 1 mL aliquot of human plasma was transferred to a 10 mL volumetric flask, and the volume was adjusted to the mark with methanol (HPLC-grade). The mixture was vortexed for 10 min to ensure complete precipitation of plasma proteins. The suspension was then centrifuged at 4000 rpm for 10 min, and the clear supernatant was carefully collected and filtered through a 0.22 μm syringe filter. The efficiency of protein removal was confirmed by the absence of turbidity in the supernatant. A 10 µL aliquot of the filtrate was applied to each TLC plate band to achieve well-resolved spots without overloading. All samples were analyzed in triplicate (*n* = 3) and stored at −20 °C for up to two weeks to maintain analyte stability prior to analysis.

## Procedures

### Chromatographic conditions

Using the autosampler, 10 µL of the standard solutions prepared as described in Sect. "Standard solutions" were applied as 6 mm wide bands on TLC plates. The bands were positioned 10 mm from the bottom edge of the plate with 10 mm spacing between adjacent bands. Chromatographic development was performed at room temperature in a tank previously saturated for 15 min with a mixture of ethyl acetate, ethanol, and ammonia (3:6.8:0.2, v/v/v) to a distance of 9 cm. The developed plates were scanned at 240 nm in absorbance mode using densitometric UV detection.

### Linearity and calibration curve

Calibration curves for CYT and GRS were constructed using a fixed concentration of FUR as an internal standard. Appropriate volumes of CYT and GRS working standard solutions were transferred into a series of 10 mL volumetric flasks, each containing a constant amount of FUR corresponding to 10 µg/band. Aliquots of 10 µL from each solution were applied to the TLC plates to obtain concentration ranges of 0.5–3.0 µg/band for CYT and GRS.

After chromatographic development and densitometric scanning, calibration curves were generated by plotting the peak area ratio of each analyte to the internal standard (CYT/FUR or GRS/FUR) against the corresponding drug concentration expressed in µg/band.

For calibration in spiked human plasma, different volumes of CYT and GRS working solutions were added to 10 mL volumetric flasks containing 1.0 mL of blank human plasma and a fixed concentration of FUR (10 µg/band). The volume was adjusted with methanol to obtain final CYT and GRS concentrations in the range of 0.5–3.3 µg/band. Sample preparation, including protein precipitation, vortex mixing, centrifugation at 4000 rpm for 10 min, and filtration through a 0.22 μm syringe filter, was performed as described in Sect. "Blank plasma samples" prior to chromatographic analysis.

## Results and discussion

Effective management of chemotherapy-induced nausea and vomiting (CINV) is crucial during leukemia treatment to ensure patient safety and adherence to the therapy [37]. The proposed thin-layer chromatographic method represents an alternative to previously reported techniques and enables the simultaneous quantification of GRS and CYT in spiked human plasma samples. This method may facilitate pharmacokinetic studies and support the therapeutic dose adjustment.

### Method optimization

Several experimental parameters were systematically investigated to achieve optimal resolution and separation. The key parameters are described below.

#### Mobile phase

**Fig. 2:**
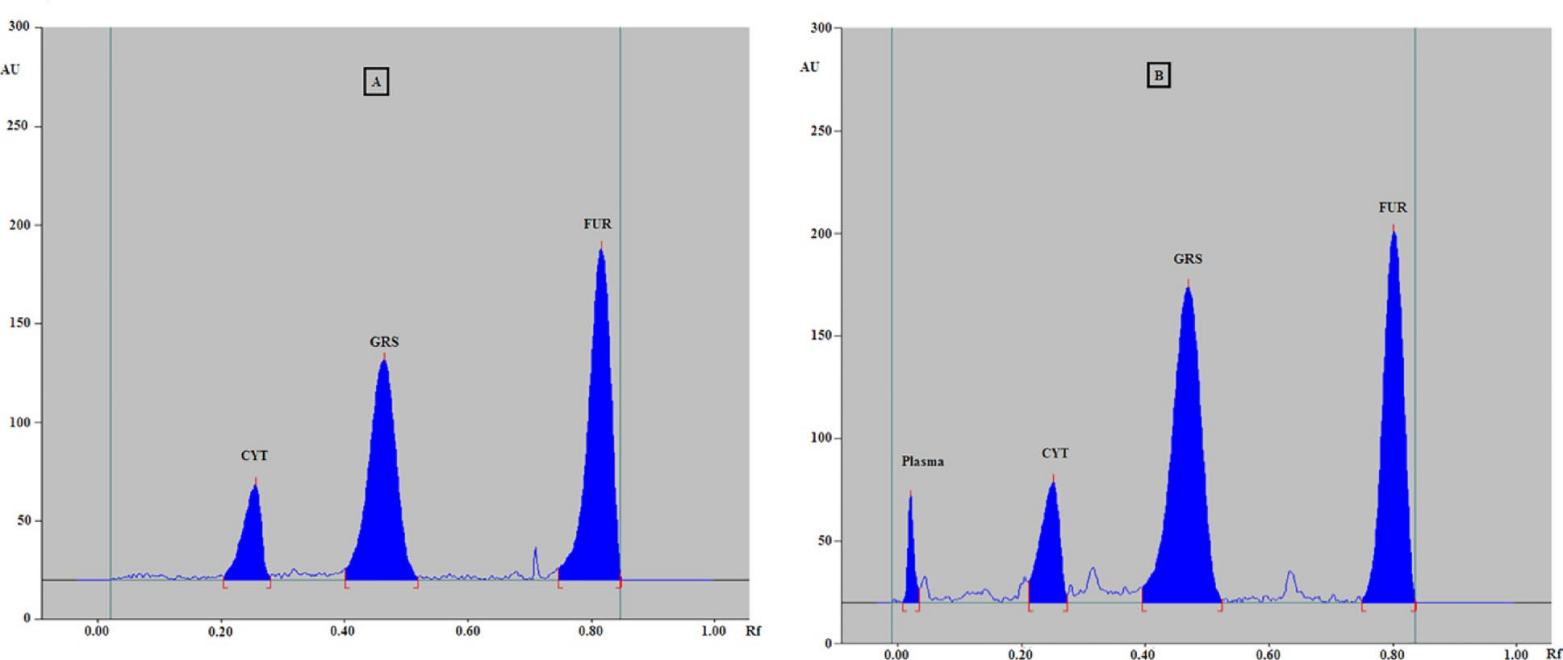
**A** Two-dimensional TLC densitogram of a mixture of cytarabine (Rf = 0.24), granisetron (Rf = 0.5), and furosemide (internal standard) (Rf = 0.8), using a developing system of ethyl acetate: ethanol: ammonia (3:6.8:0.2, by volume) and detection at 240 nm, Fig. 2. **B** Two-dimensional TLC densitogram of a mixture of plasma (Rf = 0.06), cytarabine (Rf = 0.24), granisetron (Rf = 0.5), and furosemide (internal standard) (R_f_ = 0.8) using a developing system of ethyl acetate: ethanol: ammonia (3:6.8:0.2, by volume) and detection at 240 nm

 Green solvents were evaluated for preparing the developing system for TLC. Initially, acetone: methanol (7:3, 9:1, and 6:4, v/v) and methanol: ethyl acetate (9:1, 7:3, and 6:4, v/v) mixtures were tested. In these systems, the spots of CYT, GRS, and plasma appeared close to the baseline, and a clear separation between the drugs and plasma was not achieved. Ethyl acetate-based systems improved separation compared to acetone-based systems. Using ethyl acetate and methanol in different ratios, CYT remained near the baseline, whereas GRS exhibited tailing. Replacing methanol with ethanol elevated CYT above baseline, although the GRS remained broad. The pH of the mobile phase also influenced the separation. Different volumes (0.1, 0.2, 0.3, and 0.5 mL) of ammonia solution (33%), acetic acid, and formic acid were tested individually, revealing that a basic medium (ammonia) provided the best separation of the components. The final optimized mobile phase consisted of ethyl acetate, ethanol, and ammonia (3:6.8:0.2, v/v/v). Under these conditions, plasma, CYT, GRS, and FUR exhibited Rf values of 0.06, 0.24, 0.50, and 0.80, respectively (Fig. 2). The detailed optimization parameters and results are summarized in Table S1 (Supplementary Materials).

#### Scanning wavelength

The scanning wavelengths were 225, 240, 270, 290, and 300 nm. Among these, 240 nm provided the highest sensitivity and best signal-to-noise ratio for all the studied drugs; therefore, it was selected for subsequent analysis.

#### The internal standard

The use of an internal standard is essential to compensate for potential analyte losses during sample preparation and analysis. Several compounds, including acetaminophen, dapoxetine, paracetamol, ketoprofen, caffeine, and furosemide, were evaluated as potential internal standards. Furosemide was selected as the internal standard due to its suitable chromatographic behavior, good resolution from the studied drugs, and absence of interference with their peaks.

### Method development

Calibration curves were constructed for each drug by plotting the peak area ratio of each drug to the internal standard (peak area of drug/peak area of FUR) against the corresponding concentration in µg/band, following the chromatographic procedures described in Sect. "Chromatographic conditions".

### Method validation

The proposed method was validated for linearity, range, accuracy, precision, selectivity, specificity, and stability in accordance with the U.S. Food and Drug Administration’s “Guidance for Industry: Bioanalytical Method Validation” [38].

#### Calibration curves

 Calibration ranges for the studied drugs were established using pure standard solutions and spiked human plasma samples. In spiked plasma, CYT and GRS exhibited concentration ranges of 0.5–3.3 µg/band, while in pure standard solutions, the ranges were 0.5–3.0 µg/band for both drugs. Linear regression analysis confirmed the linearity of the method, as summarized in Table S2.

#### Regression equations

The concentrations of the drugs under investigation were calculated using the corresponding regression equations, and the results are displayed in Table S2. The calculated regression equations were found to be.

$${\text{Y plasma}}\,{\mathrm{CYT}} = {\text{ }}0.{\text{2441X }} + {\text{ }}0.0{\mathrm{758}}~~~~~~~~~~~~~~{\text{r }} = {\text{ }}0.{\mathrm{99995}}$$

$${\text{Y plasma}}\,{\mathrm{GRS}} = {\text{ 1}}.0{\text{47X }} + {\text{ }}0.{\mathrm{2418}}~~~~~~~~~~~~~~~\,\,\,~{\text{r }} = {\text{ }}0.{\mathrm{99992}}$$

$${\text{Y pure}}\,{\mathrm{CYT}} = {\text{ }}0.{\text{3912X }} - {\text{ }}0.0{\mathrm{2}}0{\mathrm{5}}~~~~~~~~~~~~~~~~~~~{\text{r }} = {\text{ }}0.{\mathrm{99997}}$$

$${\text{Y pure}}\,{\mathrm{GRS}} = {\text{ 1}}.{\text{4329X }} - {\text{ }}0.{\mathrm{19}}0{\mathrm{2}}~~~~~~~~~~~~~~~~~~~\,{\text{r }} = {\text{ }}0.{\mathrm{99996}}$$

where Y is the peak area ratio, X is the concentration in µg/band, and r is the correlation coefficient.

#### Accuracy

The concentrations of CYT and GRS were determined by applying the chromatographic procedure described in Sect. "Chromatographic conditions" and using the previously established regression equations. Various concentrations within the respective linear ranges were analyzed to assess the accuracy of the proposed method. The mean recoveries for each drug were calculated and are presented in Table S2. The obtained results demonstrated high accuracy, confirming the reliability of the method.

#### Precision

Intraday precision was assessed by analyzing three replicates of CYT and GRS at concentrations of 0.5, 1.3, 1.8, 3.0, and 3.3 µg/band on the same day. The results were expressed as the percent relative standard deviation (% RSD). Interday precision was determined by analyzing three replicates of each concentration on three separate days. The obtained % RSD values were below 15% for all concentrations, indicating an acceptable precision in accordance with FDA guidelines (Table 1).

**Table 1:** Intraday and interday precision and accuracy of lower LQC (LLQC), low quality control (LQC), mid quality control (MQC), high quality control (HQC) and ULOD of spiked plasma sample

	Concentration (µg/band)		Intra – day		Inter – day	
			Recovery	%RSD	Recovery	% RSD
Cytarabine	LLQC	0.5	99.304	1.65	100.123	2.165
	LQC	1.3	100.904	0.625	100.379	1.416
	MQC	1.8	99.959	0.455	100.414	1.133
	HQC	3	100.396	0.272	100.123	0.682
	ULOD	3.3	99.711	0.249	99.959	0.621
Granisetron	LLQC	0.5	100.134	0.381	100.134	0.954
	LQC	1.3	101.281	0.689	99.909	1.392
	MQC	1.8	100.616	0.105	100.545	0.371
	HQC	3	99.815	0.178	99.815	0.336
	ULOD	3.3	100.331	0.148	99.945	0.631

#### Selectivity

The selectivity of the method was evaluated to determine whether plasma constituents interfered with CYT, GRS, or FUR detection. Plasma samples were prepared under the conditions described previously, and the results illustrated in Fig. 2 confirmed that no interference from the plasma matrix was observed at the Rf values of the studied compounds.

#### Extraction recovery

 The extraction recovery of CYT, GRS, and FUR from plasma was evaluated by comparing the mean peak areas obtained from triplicate analyses of three concentrations of each pure drug in methanol with those of the same concentrations in the spiked plasma samples. The tested concentrations of CYT and GRS were 1.3, 1.8, and 3.0 µg/band. The detailed results are presented in Table S3 (Supplementary Material).

#### Drug stability in biological fluids

The stability of a drug in a biological matrix depends on several factors, including the properties of the matrix, container system, chemical characteristics of the drug, and storage conditions. The stability of CYT and GRS in spiked human plasma was assessed and expressed as percent recovery (% recovery ± RSD).

- Benchtop stability

Three aliquots of low, medium, and high concentrations of spiked plasma samples were thawed and maintained at room temperature (25 °C) for 6 h at the beginning of the day. The stability of the processed samples, including the time spent in the autosampler, was also evaluated. For this assessment, three replicates were prepared and stored at room temperature until the end of the day. The results indicate that CYT and GRS remained stable throughout the analysis. Detailed data are provided in Table S4 (Supplementary Material).

- Freeze-thaw stability

The freeze–thaw stability of CYT and GRS in spiked plasma was evaluated over three cycles. Three aliquots at low, medium, and high concentrations were frozen at −20 °C for 24 h and subsequently thawed at room temperature (25 °C). This freeze–thaw procedure was repeated three times for each sample. The results indicated that the sample concentrations did not change significantly after three cycles, demonstrating good freeze–thaw stability (detailed data are provided in Table S4, Supplementary Material).

#### Greenness assessment profile

Recently, the greenness of chromatographic methods has attracted considerable attention. Analytical techniques involve multiple factors that may impact human health and the environment; therefore, various metric systems have been developed to evaluate different aspects of analytical sustainability. In this study, two greenness assessment tools, the Modified Green Analytical Procedure Index (MoGAPI) and the Analytical GREEnness (AGREE) metric, were applied to evaluate and compare the proposed TLC method. The results of the eco-friendliness assessment are presented in Table 2.

**Table 2:** Greenness assessment of the developed TLC- Densitometric method using two metric systems (MoGAPI, and AGREE)

Mobile phase	MoGAPI	Agree
Ethyl acetate, ethanol, and ammonia (3:6.8:0.2, by volume)		

The AGREE metric evaluates compliance with the 12 principles of green analytical chemistry by assigning a score to each principle and calculating an overall greenness score. This tool mainly focuses on parameters related to energy consumption and waste generation and overall methodological design. The developed TLC method achieved an overall AGREE score of 0.61. These results indicate that the proposed method has a minimal impact on human health and the environment.

 The Modified Green Analytical Procedure Index (MoGAPI) was applied to provide both visual assessment and quantitative scoring of method greenness. Sample preparation, reagent usage, instrumentation, and waste factors were evaluated using the MoGAPI program [39], which produced a numerical score and a color-coded pictogram representation. These results highlight the importance of employing multiple assessment tools when evaluating the greenness of an analytical procedure. Detailed information for each assessment tool is provided in Table 2. These greenness results are further integrated into the overall sustainability evaluation using the Multicolor Assessment Index, as discussed in Sect. "Multicolor assessment index".

#### Multicolor assessment index

**Fig. 3:**
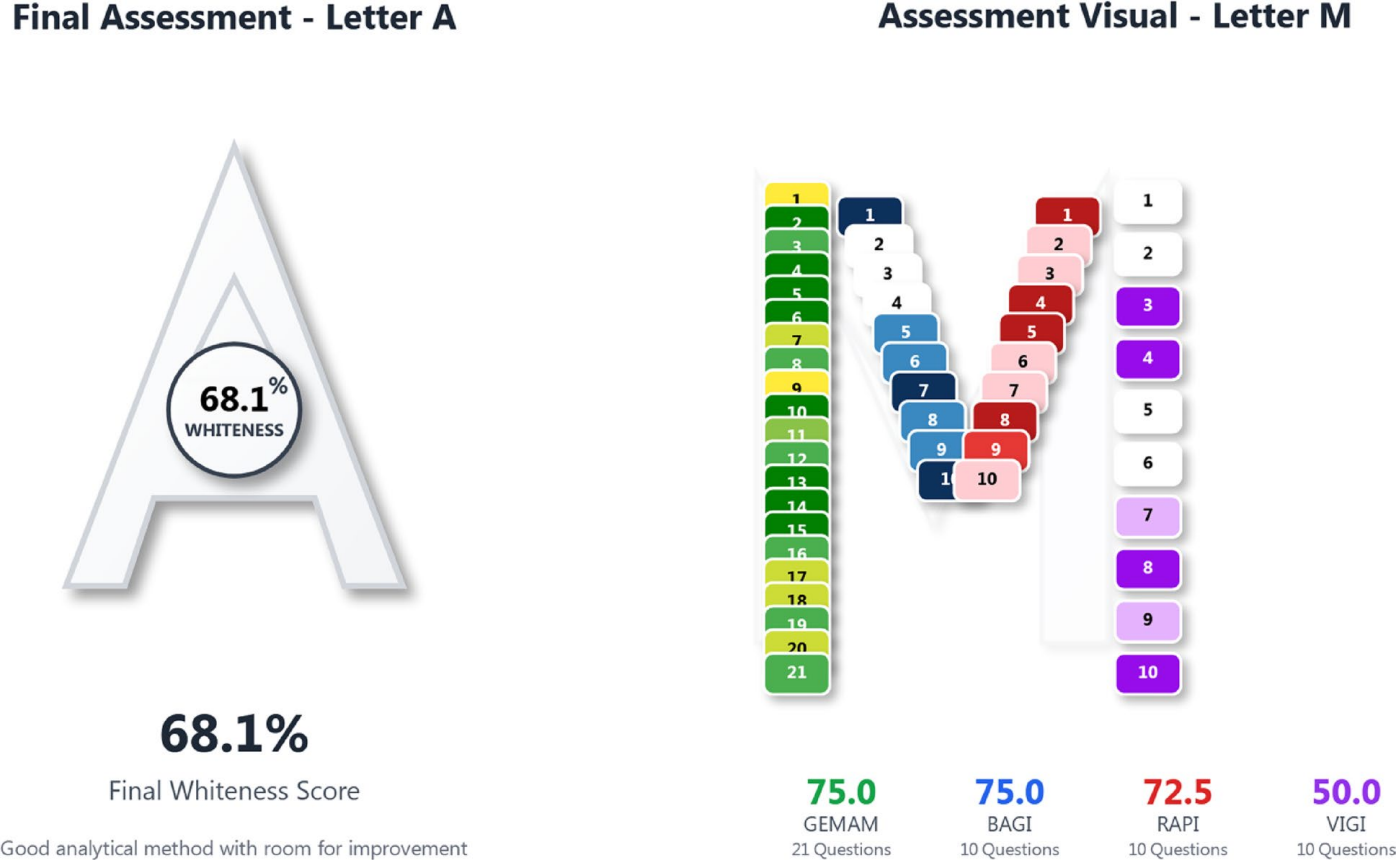
Final whiteness score (68.1%) representing the integrated balance between green principles and analytical efficiency and multi-dimensional performance profile illustrating scores for GEMAM (75%), BAGI (75%), RAPI (72.5%), and VIGI (50%)

The Multicolor Assessment (MA) [40] tool was applied to provide a comprehensive evaluation of the greenness and overall performance of the proposed method. The developed TLC method achieved an overall Whiteness Score of 68.1%. High values of GEMAM and BAGI (75.0% each) reflected environmentally favorable experimental conditions and good method applicability, while the RAPI score (72.5%) indicated acceptable analytical performance. Overall, the MA results support the environmentally friendly profile of the developed TLC method. Detailed outcomes of the Multicolor Assessment, including individual index scores and the overall Whiteness Score, are presented in Fig. 3.

#### System suitability testing parameters

 System suitability testing was performed to ensure the adequate chromatographic performance of the proposed method. Several parameters, including resolution, tailing factors, and selectivity, were calculated. The obtained values were within the recommended limits, confirming the suitability of the system for routine analysis. Detailed results are presented in Table S5 (Supplementary Material).

#### Robustness

 The robustness of the developed method was evaluated by introducing deliberate and small variations in selected experimental parameters. Minor changes in the mobile phase composition, scanning wavelength, and tank saturation time were examined. The obtained results demonstrated that these variations did not significantly affect the analytical performance of the method, confirming its robustness. The results are summarized in Table S6 (Supplementary Material).

#### Comparative and statistical study

Statistical and comprehensive comparison was conducted between the proposed TLC–densitometric method and previously reported HPLC methods [13, 27] in terms of analytical performance and environmental impact. Statistical evaluation of the results obtained for the determination of CYT and GRS in their pure forms showed that the calculated t- and F-values were lower than the corresponding theoretical values, indicating no statistically significant differences between the proposed and reported methods with respect to accuracy and precision (Table 3).

**Table 3:** Statistical comparison of the proposed TLC method with reported chromatographic methods

Items	Cytarabine	Granisetron	Reported method [13, 27]	
			Cytarabine	Granisetron
Mean	99.849	99.958	100.145	100.489
SD	1.009	0.462	0.903	1.038
N	6	6	6	6
Variance	1.019	0.251	0.815	1.078
Student t test (2.228)	0.536	1.074		
F value (5.050)	1.250	4.300		
*figures between parenthesis represent the corresponding tabulated values of t and F at P= 0.05				

13. Murthy, V.S., et al., Development and validation of RP-HPLC method for estimation of cytarabine in bulk and pharmacutical dosage forms. International Journal of Pharmaceutical Sciences and Research, 2013. 4 (12): p. 4573 - 4589.

27. Heda, A., et al., Development and validation of RP-HPLC method for simultaneous determination of granisetron and dexamethasone. Indian Journal of Pharmaceutical Sciences, 2011. 73 (6): p. 696.

In addition to demonstrating statistical equivalence, the proposed TLC–densitometric method exhibited several practical advantages. These included a wider linearity range and higher sample throughput, achieved through the simultaneous analysis of multiple samples on a single TLC plate. From an environmental perspective, the proposed method significantly reduced solvent consumption to less than 1 mL per sample and avoided the use of hazardous organic solvents, such as acetonitrile and triethylamine, which are commonly employed in the reported HPLC methods. The environmentally favorable profile of the developed method was further supported by quantitative greenness assessment using multiple evaluation tools, including MoGAPI, AGREE, and White Analytical Chemistry (WAC) metrics. Additionally, high Whiteness-related scores obtained from BAGI, RAPI, and VIGI indices highlighted the suitability of the proposed method for sustainable bioanalytical applications (Table 4).

**Table 4:** Comparative assessment of the proposed TLC method with reported chromatographic methods

Parameters	Proposed TLC method	Ref [13] (HPLC)	Ref [27] (HPLC)
Analytes	Cytarabine and Granisetron	Cytarabine	Granisetron and Dexamethasone
Matrix	Spiked Human Plasma	Bulk and Dosage forms	Bulk and Dosage forms
Linearity Range	0.5–3.3 µg/band	10–50 µg/mL	1–10 µg/mL
Solvent Consumption	Extremely Low (< 1 mL/sample)	High (Isocratic flow)	High (Isocratic flow)
Analysis time	5 min per sample	10 min per injection	8 min per injection
Sample Throughput	High (Multiple samples/plate)	Low (One sample/run)	Low (One sample/run)
Environmental Safety	Green solvents (Ethanol/ethyl acetate)	Acetonitrile (non-green) buffer (ammonium acetate) (borderline)	Acetonitrile (non-green) Triethylamine (non-green)
Assessment Tools	GAPI, AGREE, BAGI, RAPI, GEMAM and VIGI	None	None

13. Murthy, V.S., et al., Development and validation of RP-HPLC method for estimation of cytarabine in bulk and pharmacutical dosage forms. International Journal of Pharmaceutical Sciences and Research, 2013. 4 (12): p. 4573–4589

27. Heda, A., et al., Development and validation of RP-HPLC method for simultaneous determination of granisetron and dexamethasone. Indian Journal of Pharmaceutical Sciences, 2011. 73 (6): p. 696

## Conclusion

In this study, a novel, green, and sensitive TLC-densitometric method was successfully developed and validated for the simultaneous determination of CYT and GRS in spiked human plasma. Compared with previously reported sequential HPLC techniques, the proposed method offers notable improvements in analytical throughput by reducing the average analysis time per sample and minimizing solvent consumption to less than 1 mL. The environmental advantages and practical applicability of the method were rigorously evaluated using a multi-metric approach, including AGREE, MoGAPI, and the comprehensive White Analytical Chemistry (WAC) assessment, with favorable scores across BAGI, RAPI, and VIGI indices. The extraction procedure is rapid, cost-effective, and provides high recoveries without interference from the plasma matrix. Consequently, the proposed method represents an efficient and sustainable alternative for therapeutic drug monitoring and pharmacokinetic studies in patients undergoing treatment for acute myeloid leukemia.

## Supplementary Information

Supplementary Material 1

## Data Availability

The datasets supporting the conclusions of this study are available from the corresponding authors upon reasonable request and are also included within the article.
