# Supplementary material for: Green TLC-densitometric method for determination of cytarabine and granisetron in spiked human plasma: optimization and whiteness assessment
Source: BMC Chem. 2026 May 19;20(1):103. doi: 10.1186/s13065-026-01821-1 (PMC13185277; doi:10.1186/s13065-026-01821-1)
Supplement: Supplementary file 1 — Supplementary Material 1 [file 13065_2026_1821_MOESM1_ESM.docx]

**Table S1. Comparison between different tested mobile phases for the separation of Cytarabine, Granisetron, and plasma.**

| **Mobile phase composition (v/v)** | **Observation / Remarks** | **Rf Cytarabine** | **Rf Granisetron** | **Resolution** |
| --- | --- | --- | --- | --- |
| **Acetone : Methanol (7:3)** | Spots remained near baseline; poor migration | 0.08 | 0.15 | Poor |
| **Acetone : Methanol (9:1)** | Partial movement; severe tailing of GRS | 0.10 | 0.18 | Poor |
| **Methanol : Ethyl acetate (9:1)** | Overlapping peaks; insufficient separation from plasma | 0.12 | 0.25 | Moderate |
| **Methanol : Ethyl acetate (6:4)** | Improved but still broad GRS spot | 0.15 | 0.38 | Moderate |
| **Ethanol : Ethyl acetate (7:3)** | Better separation; CYT slightly tailed | 0.22 | 0.48 | Good |
| **Ethyl acetate : Ethanol : Ammonia (3:6.8:0.2)** | Sharp, symmetric peaks; excellent resolution between CYT, GRS, and plasma | 0.24 | 0.50 | Excellent |

As shown in Table S1, several mobile phase systems were investigated to achieve optimum separation. Among all the tested systems, the mixture of ethyl acetate: ethanol: ammonia (3:6.8:0.2, v/v) provided sharp, symmetric, and well-resolved spots for Cytarabine, Granisetron, and plasma components. Therefore, it was selected as the optimum developing system for further studies.

**Table S2. Validation parameters of the proposed TLC method, for determination of cytarabine and granisetron (µg/band) in pure samples and spiked human plasma.**

| **Parameters** | **Pure** | | **Plasma** | |
| --- | --- | --- | --- | --- |
|  | **Cytarabine** | **Granisetron** | **Cytarabine** | **Granisetron** |
| **Calibration range (µg/band)** | 0.5 – 3 µg/band | 0.5 – 3 µg/band | 0.5 – 3.3 µg/band | 0.5 – 3.3 µg/band |
| **Slope** | 0.3912 | 1.4329 | 0.2441 | 1.047 |
| **Intercept** | 0.0205 | 0.1902 | 0.0758 | 0.2418 |
| **Correlation coefficient** | 0.99997 | 0.99996 | 0.99995 | 0.99992 |
| **Accuracy (mean ± SD)** | 99.849 ± 1.009 | 99.958 ± 0.462 | 99.975 ± 1.295 | 99.860 ± 1.026 |
| **LLOQ** |  |  | 0.5 | 0.5 |
| **ULOQ** |  |  | 3.3 | 3.3 |

**Table S3. Extraction recovery results of the studied drugs in spiked human plasma by TLC method.**

| **Analyte** | **Concentration (µg/band)** | **% Recovery** |
| --- | --- | --- |
| **Cytarabine** | 1.3 | 99.959 |
|  | 1.8 | 100.49 |
|  | 3 | 100.123 |
|  | Mean ± SD | 100.191 ± 0.272 |
| **Granisetron** | 1.3 | 100.767 |
|  | 1.8 | 99.819 |
|  | 3 | 100.197 |
|  | **Mean ± SD** | 100.261 ± 0.477 |

**Table S4. Results of stability studies of the drugs in spiked human plasma under different conditions using the proposed TLC method.**

| **Analyte** | **% Recovery** | | |
| --- | --- | --- | --- |
|  | **Concentration**  **(µg/band)** | **Three freeze thaw cycles** | **Benchtop stability** |
| **Cytarabine** | 1.3 | 102.059 | 99.854 |
|  | 1.8 | 101.249 | 98.593 |
|  | 3 | 98.894 | 99.03 |
| **Mean ± SD** | Mean ± SD | 100.734 ± 1.633 | 99.159 ± 0.646 |
| **Granisetron** | 1.3 | 100.35 | 98.881 |
|  | 1.8 | 98.758 | 99.66 |
|  | 3 | 97.99 | 100.229 |
| **Mean ± SD** | **Mean ± SD** | 99.033 ± 1.216 | 99.59 ± 0.679 |

**Table S5. System suitability testing parameters of the proposed TLC densitometric method for the determination of the proposed drugs.**

| **Parameters** | **Cytarabine** | **Granisetron** | **Furosemide** |
| --- | --- | --- | --- |
| **Capacity factors** | 1.481 | 3.148 | 5.556 |
| **Symmetry factor** | 0.9 | 1.09 | 0.97 |
| **Resolution** |  | 2.57 | 3.25 |
| **Selectivity** |  | 2.126 | 1.765 |

**Table S6. Results of robustness study of the proposed TLC method for determination of the proposed drugs.**

| Factors | **Cytarabine** | **Granisetron** |
| --- | --- | --- |
| Scanning wavelength ± 0.74 % | 0.531 | 0.442 |
| Saturation time ± 4.2 % | 0.611 | 0.559 |
| Ethanol ratio ± 2 % | 0.553 | 0.465 |
